# Meeting of the American Medical Association

**Published:** 1893-07

**Authors:** 


### *EDITORIAL.*

The office of THE JOURNAL is in room 330 Equitable Building.
Address communications relating to the Editorial Department to DR. L. B. GRANDY, Atlanta, Ga., Box 431.
Business communications should be addressed to DR. M. B. HUTCHINS, Atlanta, Ga., Box 431.
All remittances should be made payable to "ATLANTA MEDICAL AND SURGICAL JOURNAL."
Articles for publication should reach this office not later than the 15th instant.
The editors are not responsible for views expressed by contributors.

### THE MEETING OF THE AMERICAN MEDICAL ASSOCIATION.

The late meeting of the Association at Milwaukee was well attended by medical men from all over the country. We regret that the attendance from the South was rather meager. The meeting of the Association was preceded by that of the Medical Editors. Before this Association Mr. Ernest Hart, editor of the *British Medical Journal,* delivered a learned and excellent address on "Medical Journalism."

Before the general association Mr. Hart read an exhaustive paper on "Cholera; Where it Comes from and how it is Propagated." The Address in General Medicine was delivered by Dr. H. A. Hare, of Philadelphia, in which he gave a review of some late practical advances in medicine and therapeutics. Dr. H. H. Mudd, of St. Louis, delivered the Address in Surgery on Surgical Problems, in which he discussed the present status of the drainage tube, the surgical relief of hernia and the treatment of appendicitis. The Address in State Medicine was read by Dr. Walter Wyman, Surgeon-General of the Marine Hospital Service, on the Extinction of Contagious Diseases. He spoke of the gradual disappearance of yellow fever, smallpox and typhus fever, and said that we were warranted in hoping that means would be discovered for the extermination of cholera.

The Committees on Revision of the Constitution and Revision of the Code made preliminary reports, but asked to be given until the next meeting to make their full reports. A committee appointed at the previous meeting reported in favor of petitioning Congress to create a Department and a Secretary of Public Health.

Dr. J. McFadden Gaston and Dr. F. W. McRae were in attendance upon the Association from Atlanta. The former presented a paper on the "Present Status of Thoracic Surgery." We are pleased to note that our *Journal* colleague, Dr. McRae, was made Secretary of the Surgical Section of the Association. It is an honor which Dr. McRae thoroughly deserved.

The Association will meet next year in San Francisco the first Tuesday in May. The officers for the ensuing year are: President, Dr. James F. Hibberd, of Indiana; Vice-Presidents, Dr. Jno. A. Wyeth, New York; Dr. I. N. Love, Missouri, and Dr. O. B. Wingate, Wisconsin; Secretary, Dr. William B. Atkinson, Philadelphia; Judicial Council, Dr. X. C. Scott, Ohio; Dr. G. W. Stone, Marine Hospital Service; Dr. J. H. Murphy, Minnesota; Dr. J. McF. Gaston, Georgia; Dr. T. A Foster, Maine, and Dr. I. N. Quimby, New Jersey.

